# Antifatigue Effect of Luteolin-6-C-Neohesperidoside on Oxidative Stress Injury Induced by Forced Swimming of Rats through Modulation of Nrf2/ARE Signaling Pathways

**DOI:** 10.1155/2017/3159358

**Published:** 2017-05-15

**Authors:** Fang-fang Duan, Ying Guo, Jing-wan Li, Ke Yuan

**Affiliations:** ^1^College of Jiyang, Zhejiang Agriculture and Forestry University, Zhu'ji 311800, China; ^2^College of Forestry and Biotechnology, Zhejiang Agriculture and Forestry University, Lin'an 311300, China; ^3^College of Life Science, Zhejiang Chinese Medical University, Zhejiang 310053, China

## Abstract

Luteolin-6-C-neohesperidoside (LN) is a flavonoid isolated from moso bamboo leaf. This study was performed to evaluate the antifatigue effect of LN on a rat model undergoing the weight-loaded forced swimming test (FST). Briefly, male Sprague-Dawley rats (20–22 weeks old) were forced to undertake exhaustive swimming every other day for 3 weeks. Each swimming session was followed by the administration of distilled water, LN (25–75 mg/kg), or ascorbic acid (100 mg/kg) 1 h later. Oral administration of LN significantly improved exercise endurance; normalized alterations in energy metabolic markers; and decreased serum lactic acid, lactate dehydrogenase, and blood urea nitrogen levels of rats that underwent FST. Moreover, LN enhanced the activities of antioxidant enzymes and antioxidant capacity, as measured by enzyme activity assays, RT-PCR, and Western blotting, as well as decreasing the levels of proinflammatory cytokines such as tumor necrosis factor-*α*, interleukin-1*β* (IL-1*β*), and IL-6 and increasing the level of anti-inflammatory (IL-10) in the liver and skeletal muscle. These results suggested that LN reduces both physical and mental effects of chronic fatigue, probably by attenuating oxidative stress injury and inflammatory responses in the liver and skeletal muscle. This study thus supports the use of LN in functional foods for antifatigue and antioxidant effects.

## 1. Introduction

Chronic fatigue is characterized by persistent tiredness that is not relieved by rest and that is always worsened by physical or mental activity [1]. Physical fatigue is caused by excessive exercise, and long-term accumulated physical fatigue can lead to a series of fatigue-related syndromes, including cognitive problems, neurocognitive dysfunction, sleep disturbances, gastrointestinal symptoms, and postexertional malaise [2]. Approximately 10% of the general population around the world suffers from chronic fatigue, which has significant detrimental effects on the ability to work and the quality of daily life [3, 4]. Despite the prevalence of this condition, the etiology of fatigue has not been fully elucidated. In view of ongoing research on chronic fatigue, it is emphasized that the pathogenesis of fatigue is associated with energy metabolism and immune and endocrine systems, as well as inflammatory response and dysfunction of the antioxidant defense system [5–7]. Among these potential factors, oxidative stress plays the most important role in the etiology of fatigue. Increased oxidative stress due to increased secretion of pro-oxidants and reactive oxygen species (ROS), as well as lipid peroxidation, can be observed in humans with chronic fatigue syndrome [8, 9]. Meanwhile, oxidative stress was shown to stimulate the inflammatory response by increasing the release of proinflammatory cytokines such as tumor necrosis factor-*α* (TNF-*α*), interleukin-6 (IL-6), and IL-1*β* in patients with chronic fatigue [10].

Accordingly, antioxidant treatment might be one of the most valuable therapeutic approaches for fatigue, although there are few effective pharmacological drugs or therapies that can successfully eliminate patient fatigue [11]. Therefore, it is important to find natural compounds with antioxidative activities and/or to develop safe and effective antifatigue functional foods to improve exercise capacity, postpone fatigue, and accelerate the elimination of fatigue in patients [12, 13]. Previous studies reported that phytochemicals such as antioxidants absorbed from tea, fruit, and medicinal plants could not only reduce free radical formation and scavenge free radicals but also possess considerable antifatigue activity [14]. Flavonoids from plants are considered to be one of the most effective groups of phytochemicals due to their wide range of pharmacological activities, such as antioxidative, anti-inflammatory, immunomodulatory, antidiabetic, and antitumor effects [15–18]. Flavonoids have also been widely studied as a new group of natural antifatigue substances; many studies have revealed that flavonoid supplementation enhanced the endurance exercise performance of fatigued rats by reducing their muscle fatigue [19, 20].

**Figure 1:**
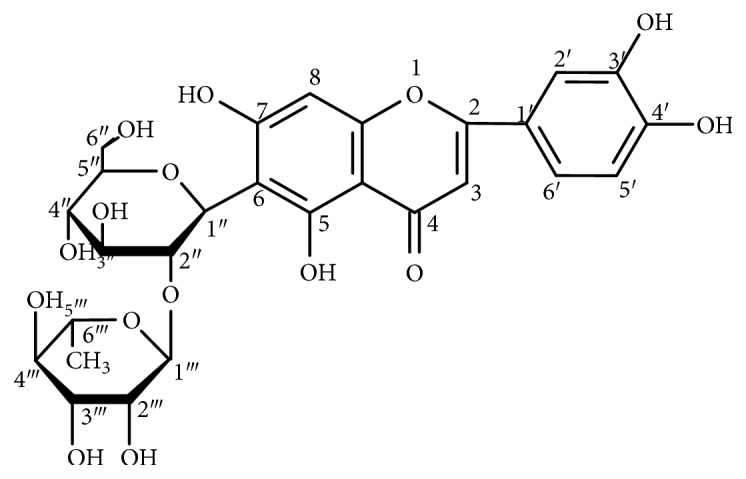
Chemical structure of luteolin-6-C-neohesperidoside (LN).

Moso bamboo (*Phyllostachys heterocycla* cv. pubescens), belonging to the family Gramineae, is one of the most representative economically important bamboo resources. It is widely distributed in the tropics and subtropics in China and has significant economic and cultural value through its use for paper, food, and traditional Chinese medicine [21, 22]. In folk medicine, bamboo leaves have been used to treat many diseases via both direct medical and indirect nutritional effects. Bamboo leaves are rich in polysaccharides, amino acids, alkaloids, polyphenols, and natural antioxidant flavonoids, such as orientin, isoorientin, vitexin, and isovitexin [23, 24]. In recent years, studies have also found that these flavonoids from bamboo leaves have excellent biological activities, such as antioxidant, antiaging, anti-inflammatory, antibacterial, antihyperlipidemia, and immunoregulatory effects [25, 26]. In our previous study, a flavonol glycoside, 5,7,3′,4′-tetrahydroxy-6-C-neohesperidose flavonol glycoside (luteolin-6-C-neohesperidoside, LN), as shown in Figure 1, isolated from moso bamboo leaf was revealed to have strong capacity for scavenging DPPH and FRAP free radicals (data not shown). To further investigate its pharmacological effects, the present study was carried out to evaluate the protective effect of LN against oxidative stress injury of organs induced by the weight-loaded forced swimming test (FST) in rats.

## 2. Materials and Methods

### 2.1. Materials and Reagents

Fresh leaves of moso bamboo were obtained from Lin'an, Zhejiang Province, and were authenticated by Prof. Lu-huan Lou, a professor of plant taxonomy at Zhejiang Agriculture and Forestry University. Samples were dried at 60°C for 24 h and then ground and passed through a 40-mesh sieve. All kits were supplied by Jiancheng Biotech. Sci. Inc., Nanjing, China. All other chemicals and reagents used were of analytical grade and obtained from Sinopharm Chemical Reagent Co. Ltd. (Shanghai, China).

### 2.2. Isolation of LN

LN was isolated from moso bamboo leaf. Briefly, about 15 kg of dried leaf powder was immersed in 15 L cold 75% ethanol for 24 h and then extracted at room temperature. The supernatant was concentrated in a rotary evaporator (RV8; IKA, Germany) at reduced pressure at 45°C. The above procedures were repeated two times with recycled ethanol (reprepare the concentration). Then, a brownish-black residue was obtained (yield 1.25 kg), which was dispersed into H_2_O (5 L) with vigorous stirring and then consecutively extracted with petroleum ether, ethyl acetate, and n-butanol for five times (2.0 L/time). These extracts were concentrated to yield a petroleum ether fraction (56 g), an ethyl acetate fraction (163 g), an n-butanol fraction (365 g), and a water fraction (246 g).

The n-butanol fraction (365 g) was subjected to column chromatography over Diaion HP-20 (10 × 130 cm) and consecutively eluted in an H_2_O-MeOH solution. All of the 40% MeOH fractions were collected together and then subjected to column chromatography over MCI Gel CHP-20, Sephadex LH-20, and silica gel, eluting with MeOH-H_2_O gradient solution. In the end, we got 13.2 g of LN from 40% MeOH solution and lyophilized them into powder for further analysis. The yield of LN is 0.088% and its pure is 98.16%.

### 2.3. Animals

Male Sprague-Dawley rats (weighing 180–220 g; 20–22 months old) were purchased from Zhejiang Academy of Medical Sciences Laboratory Animal Center [number of animal license: SCXK (Zhejiang) 2016-0613]. The rats were caged in specific pathogen-free facilities under a controlled environment [constant temperature (25 ± 2°C), humidity (45%–55%), and a constant dark/light cycle of 12 : 12 h] with food and water provided ad libitum. All procedures of animal handling and experiments were approved by the Institutional Animal Committee of Zhejiang Chinese Medical University, and all rats received care throughout the experiment in accordance with the Guide for the Care and Use of Laboratory Animals (National Institutes of Health, Bethesda, MD, USA).

### 2.4. Experimental Design

After acclimatization for one week, all animals were randomly divided into six groups (10 rats each group) as follows: normal control group (NC), model control group (MC), positive control group (ascorbic acid, 100 mg/kg, AA100), and LN-treated groups (high, 75 mg/kg, LN75; medium, 50 mg/kg, LN50; and low, 25 mg/kg, LN25). The doses of LN and ascorbic acid were decided based on our preliminary tests (data not shown) and previous studies [27]. LN and ascorbic acid were dissolved in distilled water and were orally administrated once daily for 3 weeks, 1 h before forced treadmill exercise. NC and MC were administered with the same volume of distilled water orally.

### 2.5. Weight-Loaded Forced Swimming Test (FST)

The weight-loaded FST was performed as described previously with some minor modifications [28]. The rats of the LN-administered and MC groups were subjected to FST every other day while supporting constant loads (fixed to the tail base) corresponding to 5% of their body weight for a period of three weeks. FST was carried out in a swimming tank (50 × 50 × 40 cm) with 30 cm deep water maintained at 25 ± 2°C for 1 h after oral administration of the corresponding drugs. The rats were then removed from the pool, dried with a paper towel, and returned to their original cages. The pool water was replaced after each session. Exhaustion was determined by observing the loss of coordinated movements and failure to swim. Endurance time was recorded immediately when the rat was completely exhausted and failed to return to the surface to breathe within 5 s. Animals were sacrificed under mild anesthesia immediately after the last exercise. Blood was collected from the abdominal aorta into centrifuge tubes using a heparinized syringe. Separated plasma and tissue samples of the liver and skeletal muscle were stored at −80°C until further analysis.

### 2.6. Histological Analysis of the Liver and Skeletal Muscle

Histological sections (4 *μ*m thickness) were prepared from the liver and skeletal muscle fixed in 10% buffered formaldehyde and then embedded in paraffin. Histological sections were stained with hematoxylin-eosin; stained areas were observed using an optical microscope at 100x magnification.

### 2.7. Biochemical Examination of Serum and Tissues

Serum was obtained from blood samples, and biochemical parameters in serum including blood urea nitrogen (BUN), lactate dehydrogenase (LDH), plasma lactic acid (LA), liver glycogen (LG), skeletal muscle glycogen (MG), aspartate aminotransferase (AST), and alanine aminotransferase (ALT) were determined by a spectrophotometric detection method using commercially available kits (Jiancheng Biotech. Sci. Inc., Nanjing, China). The levels of these parameters were determined in accordance with the manufacturer's protocols.

Skeletal muscle and liver tissues were homogenized to examine oxidant-related parameters, such as reactive oxygen species (ROS), malondialdehyde (MDA), superoxide dismutase (SOD), and glutathione peroxidase (GSH-Px), and inflammation-related parameters, including IL-1*β*, TNF-*α*, IL-6, and IL-10. The levels of the above biochemical parameters in the skeletal muscle and liver were measured by ELISA kits (Jiancheng Biotech. Sci. Inc.).

### 2.8. Semiquantitative RT-PCR Analysis for Nrf2 and HO-1 mRNA

Total RNAs were isolated from the liver and skeletal muscle tissues, respectively, with Trizol reagent (Sangon Biotech Co. Ltd., Shanghai, China). The mRNA of nuclear factor E2-related factor 2 (Nrf2) and heme oxygenase-1 (HO-1) was determined by semiquantitative RT-PCR analysis and normalized with *β*-actin. Briefly, 1 *μ*g of total RNA was reverse-transcribed using oligo dT and reverse transcriptase (Boya Co. Ltd., Shanghai, China). Then, cDNAs of Nrf2 and HO-1 were amplified using oligonucleotide primers (Table 1) by One-step RT-PCR kit (Takara Co., Japan). The following PCR conditions were applied: Nrf2 and HO-1, 35 cycles of denaturation at 95°C for 15 s and annealing at 60°C for 15 s extending at 72°C for 1 min, and *β*-actin, 25 cycles of denaturation at 94°C for 30 s and annealing at 60°C for 30 s extending at 72°C for 30 s. The PCR products were subjected to horizontal electrophoresis on 0.8% agarose gels, and images were captured in a Bio-Rad ChemiDoc imaging system (Hercules, CA, USA).

### 2.9. Western Blot Analysis for Nrf2 and HO-1 in the Liver and Skeletal Muscle

The liver and skeletal muscle were, respectively, homogenized in RIPA Lysis Buffer and centrifuged at 14,000 rpm (30 min, 4°C) to obtain total protein. Then, the supernatant protein concentrations in the extracts were measured using the BCA Protein Assay Kit (Aidlab Biotechnologies Co. Ltd., Beijing, China). For Western blot analysis, equal amounts of protein (50 *μ*g per lane) were loaded in the wells of 12% polyacrylamide gels. After the electrophoretic run, proteins were electrotransferred onto polyvinylidene fluoride (PVDF) membranes (Millipore, Marlborough, MA, USA). The membrane was incubated for 3 h in blocking buffer (1 × TBS, 0.1% Tween 20, and 4% nonfat milk) at room temperature and then overnight in the same buffer containing the primary antibodies against Nrf2 (1 : 1000), HO-1 (1 : 1000), and *β*-actin (1 : 1500) (Boster Biological Technology Ltd., Wuhan, China). The membrane was washed three times for 5 min and incubated for 2 h at 4°C with HRP-conjugated secondary antibodies (anti-rabbit and anti-rat) (Boster Biological Technology Ltd., Wuhan, China). Proteins were detected using an enhanced chemiluminescence detection system (Amersham Pharmacia, Piscataway, NJ, USA).

### 2.10. Statistical Analyses

All data are expressed as mean ± standard deviation of at least three separate experiments. Significant differences between the groups were determined using the one-way ANOVA. Tukey's HSD *t*-test was used for multiple comparisons. All statistical analyses were performed using IBM SPSS software, ver. 20.0 (SPSS Inc., Chicago, IL, USA). Statistical significance was regarded at *P* < 0.05.

## 3. Results

### 3.1. Effects of LN on Enhanced Swimming Ability

**Figure 2:**
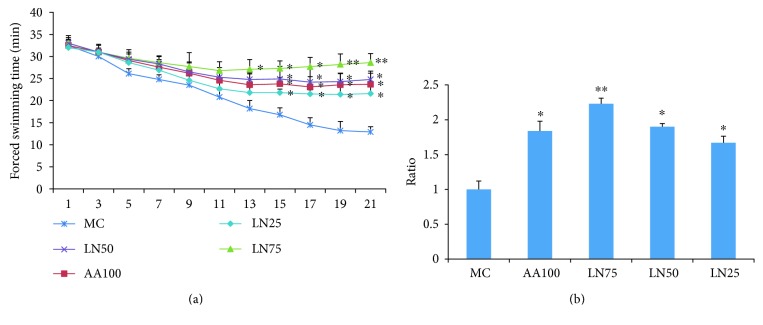
Effects of LN on the weight-loaded swimming time (a) and the ratio of exhausted swimming time (b) in FST rats. Values are the means ± SD (*n* = 10). ^∗^*P* < 0.05, ^∗∗^*P* < 0.01, and ^∗∗∗^*P* < 0.001 compared with MC (model control group).

After AA100 and LN supplementation for 3 weeks, the swimming ability of the rats was increased when compared with that of the MC group (Figure 2). Significant dose-dependent increases were observed in the groups of LN (75 mg/kg, *P* < 0.01; 50 and 25 mg/kg, *P* < 0.05).  Figure 2(b) shows that the increased ratio of exhausting swimming time of each treatment group (LN75, LN50, and LN25) after LN administration was 123.34%, 90.65%, and 67.68%, respectively.  Figure 2(a) shows that the swimming time to exhaustion in the groups of LN75, LN50, and LN25 was 28.6 ± 2.1, 24.8 ± 1.9, and 21.6 ± 1.2 min, respectively, longer than that (12.9 ± 1.2 min) in the MC rats on the last day of the FST.

### 3.2. Effects of LN on Serum Biochemical Parameters

The serum levels of LA, LDH, and BUN of all studied groups are shown in Table 2. The chronic forced exercise group exhibited significantly increased levels of LA (*P* < 0.001) and LDH (*P* < 0.01) in serum relative to those in the NC group. These effects were significantly attenuated following LN treatment (*P* < 0.001 for both 75 and 50 mg/kg LA, *P* < 0.01 for 25 mg/kg LA; *P* < 0.001 for 75 mg/kg LDH, *P* < 0.05 for both 50 and 25 mg/kg LDH, relative to those in the model control group; Table 2). The level of BUN in the MC group was significantly increased 1.8-fold compared with that in the NC group. However, LN intervention prevented the release of BUN levels in a dose-dependent manner (*P* < 0.01). Moreover, there was no obvious difference of BUN levels between the NC group and the LN75 group (*P* < 0.05).

### 3.3. Effects of LN on Glycogen Content of the Liver and Skeletal Muscle

The effects of LN on glycogen content in the liver and skeletal muscle are shown in Table 2. Compared with those in the MC group, the levels of LG and MG were significantly increased in the LN administration groups (*P* < 0.05). In particular, upon the administration of a high dose of LN (75 mg/kg), liver glycogen and muscle glycogen were increased 2 times and 1.5 times compared with those in the MC group, respectively.

### 3.4. Effects of LN on ALT and AST

In the present study, we determined the levels of ALT and AST in the serum of all rats. As shown in Table 2, exposure to the FST led to increases in ALT and AST levels in serum compared with those in the NC group. All of these effects were blocked by the administration of LN (*P* < 0.01 for 75 mg/kg ALT, *P* < 0.05 for both 50 and 25 mg/kg ALT; *P* < 0.01 for both 75 and 50 mg/kg AST, *P* < 0.05 for 25 mg/kg AST, relative to those in the control group).

### 3.5. Effects of LN on Liver and Skeletal Muscle Pathology

**Figure 3:**
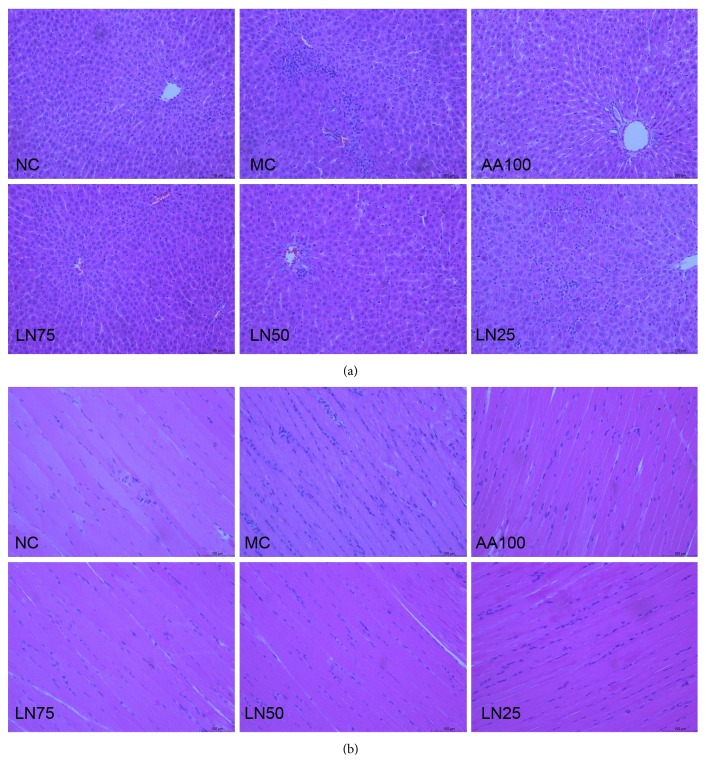
Effects of LN on the histological appearance of the liver (a) and skeletal muscle (b) are shown by photomicrographs. Representative histological sections of the liver and skeletal muscle were stained by hematoxylin and eosin (magnification ×100).

The appearances of the liver and skeletal muscle in histological analyses in the rats are shown in Figure 3. The cellular morphology of the liver and skeletal muscle in the NC group remained normal, while all exercise group samples revealed some abnormalities, such as congestion and edema along with inflammatory cell activity in the liver and skeletal muscle. Moreover, significant disordered smooth muscle cells and an increasing number of foam cells could also be observed. Compared with those in the model group, the swelling of liver and skeletal muscle cells and the degree of inflammatory cell infiltration were significantly reduced in the LN-treated groups and the AA100 group, in a dose-dependent manner.

### 3.6. Effects of LN on the Levels of Oxidant-Related Parameters in the Liver and Skeletal Muscle

**Figure 4:**
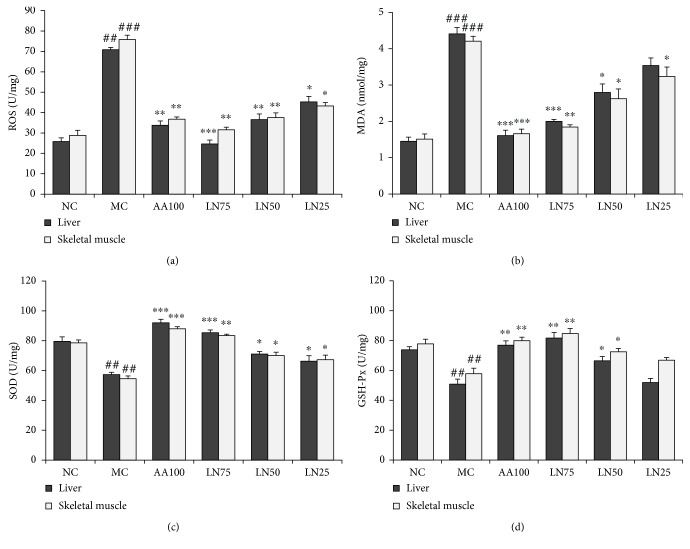
Effects of LN on stress-related production of oxidants and antioxidants in the liver and skeletal muscle. Levels of ROS (a), MDA (b), SOD (c), and GSH-Px (d) in the liver and skeletal muscle were determined by ELISA. *^##^P* < 0.01 and *^###^P* < 0.001 compared with NC (normal control group); ^∗^*P* < 0.05, ^∗∗^*P* < 0.01, and ^∗∗∗^*P* < 0.001 compared with MC (model control group).

As shown in Figure 4, compared with those in the NC group, the levels of ROS and MDA increased significantly in the liver (*P* < 0.001) and skeletal muscle (*P* < 0.01) and the activities of SOD and GSH-Px were all significantly decreased in the two organ tissues (*P* < 0.01) in the MC group. In contrast, the exhaustive exercise-induced higher ROS and MDA levels and lower antioxidant enzyme activities in the liver and skeletal muscle were significantly attenuated by LN supplementation compared with those in the MC group (*P* < 0.01 for 75 mg/kg; *P* < 0.05 for 50 and 25 mg/kg).

### 3.7. Effects of LN on the Levels of Cytokines in the Liver and Skeletal Muscle

**Figure 5:**
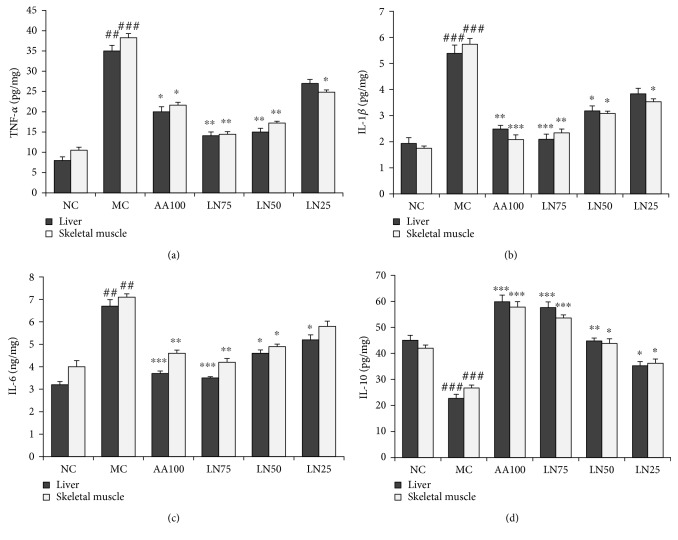
Effects of LN on pro- and anti-inflammatory cytokine levels in the liver and skeletal muscle. Levels of TNF-*α* (a), IL-1*β* (b), IL-6 (c), and IL-10 (d) in the liver and skeletal muscle were determined by ELISA. *^##^P* < 0.01 and *^###^P* < 0.001 compared with NC (normal control group); ^∗^*P* < 0.05, ^∗∗^*P* < 0.01, and ^∗∗∗^*P* < 0.001 compared with MC (model control group).

The levels of TNF-*α*, IL-1*β*, and IL-6 in the MC group were significantly increased compared with those in the NC group (all *P* < 0.01 in both the liver and skeletal muscle), while the production of IL-10 was substantially decreased in these two organs (*P* < 0.001). The administration of LN significantly ameliorated the changes in the levels of TNF-*α*, IL-1*β*, IL-6, and IL-10 in a dose-dependent manner to near the baseline (Figure 5). Similar effects on inflammatory cytokine levels were seen in response to ascorbic acid.

### 3.8. Effect of LN on the Expression of HO-1 and Nrf2 in the Liver and Skeletal Muscle

**Figure 6:**
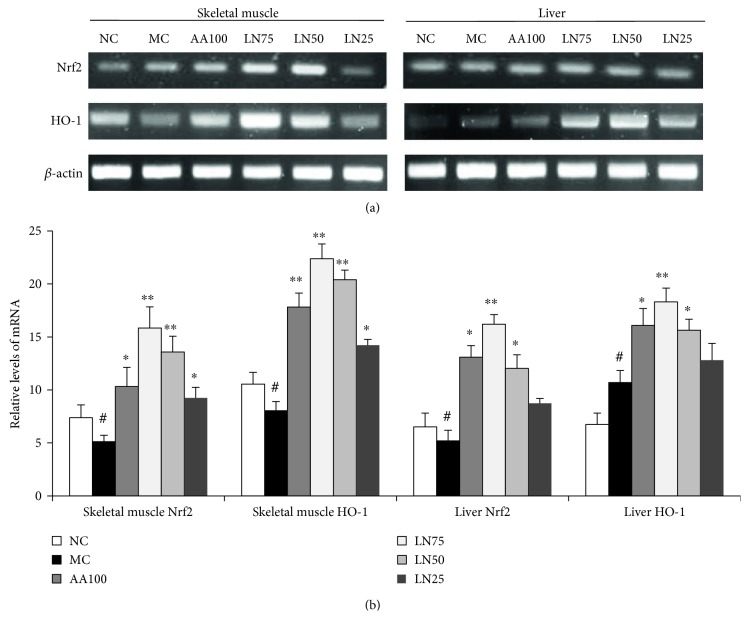
Effects of LN on the mRNA of Nrf2 and HO-1 in the liver and skeletal muscle of FST rats. (a) Representative PCR bands. (b) Relative levels of mRNA. Data are expressed as means ± SD (*n* = 10). ^#^*P* < 0.05 compared with NC (control group); ^∗^*P* < 0.05, ^∗∗^*P* < 0.01, and ^∗∗∗^*P* < 0.001 compared with MC (model control group).

**Figure 7:**
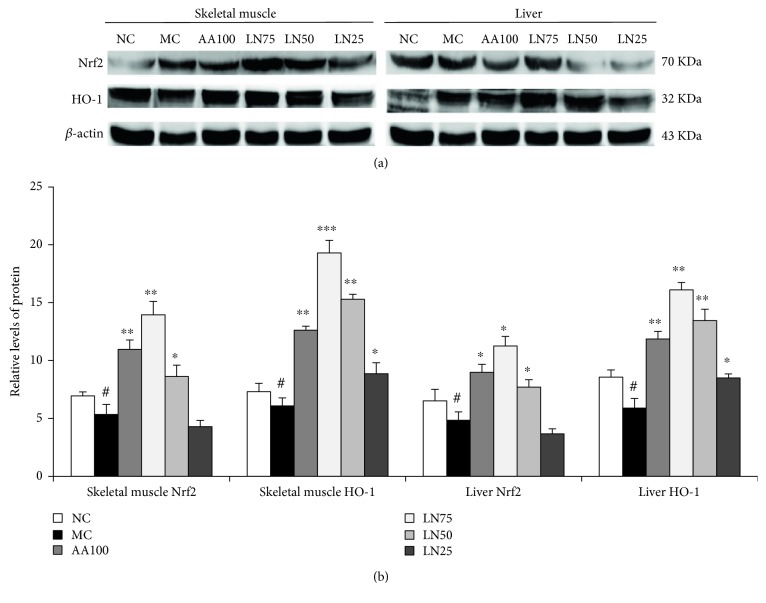
Effects of LN on antioxidant-related proteins. Levels of Nrf2 and HO-1 in the liver and skeletal muscle of FST rats were determined by Western blot (a). Respective protein adducts were quantified using ImageJ software (b). Data are expressed as means ± SD (*n* = 10). ^#^*P* < 0.05 compared with NC (control group); ^∗^*P* < 0.05, ^∗∗^*P* < 0.01, and ^∗∗∗^*P* < 0.001 compared with MC (model control group).

The expression of HO-1 mRNA and Nrf2 mRNA was hardly detected in both the liver and skeletal muscle of the MC group, while it significantly increased in the groups administered by LN (*P* < 0.05) (Figure 6). In addition, the regulatory effects of LN were found to increase in a concentration-dependent manner. Moreover, the protein expression levels of HO-1 and Nrf2 in both the liver and skeletal muscle of the MC group were generally reduced compared with those in the NC group (*P* < 0.05) (Figure 7). After LN treatment, the expression of these proteins was significantly increased in both the liver and skeletal muscle when compared with that in the MC group (*P* < 0.01 for 75 mg/kg and *P* < 0.05 for 50 mg/kg in the liver; *P* < 0.01 for 75 mg/kg and 50 mg/kg, *P* < 0.05 for 25 mg/kg in the skeletal muscle).

## 4. Discussion

Living in high-paced, modern societies can lead to increased stress, which has been identified as a factor contributing to chronic physical and mental fatigue [29]. According to traditional Chinese medicine, chronic fatigue is caused by disorders of Qi (circulating energy) and blood, which can induce the dysfunction of the liver, spleen, and kidney. LN is a flavonol glycoside isolated from moso bamboo leaf, and flavonoid is a potent antioxidant used to treat various kinds of disorders related to fatigue, such as hypertension, coronary heart disease, tumor, and diabetes, as well as inflammation [30, 31]. Here, we investigated the antifatigue activity of LN by evaluating muscle and liver functional activities, as analyzing both the phenotypic and molecular responses to LN treatment in an experimental model of FST for the first time.

The FST represents a valid animal model for screening the antifatigue potency of various bioactive compounds [32]. Swimming to exhaustion leads to both physical and mental fatigue, and the duration of swimming to exhaustion indicates the degree of fatigue [33]. An improvement of exercise endurance is the most powerful representation of an antifatigue effect. In the study, a three-week swimming model of rats was carried out. The results demonstrated that LN treatment prolonged the exhausting time of rats, indicating that LN possesses an antifatigue effect (Figure 2).

Glycogen is a secondary form of long-term energy storage, which is related to the endurance capacity of the body [34]. It acts as an energy reserve that can be quickly mobilized to meet a sudden need for blood glucose during exercise and to maintain the blood glucose level within the physiological range [35]. Studies revealed that significant consumption of glycogen in both the liver and skeletal muscle during exhaustive exercise is responsible for elevated fatigue [36]. Thus, the depletion of glycogen content is an integral factor in the development of fatigue. In our study, the levels of LG and MG were increased in LN-treated groups when compared with those in the MC group (Table 2). Such an effect might become advantageous during ameliorated physical fatigue and prolonged exercise.

Simple physical exercise begins with an increase in aerobic muscular activity, while intensive exercise leads to switching over to anaerobic metabolism, which results in the conversion of LDH to LA [37]. LA is a common marker of skeletal muscle fatigue, and excess LA inhibits the contractile activity as well as glycolysis in muscle tissue [38]. As with LA production, similar changes in serum LDH, a key enzyme required for lactate production, were revealed by the tests. Increased levels of LDH are considered an indication of cellular necrosis and tissue damage [39]. BUN is a byproduct of energy metabolism, which represents normal kidney function, and many factors such as fatigue and stress can alter BUN levels. There is a positive correlation between BUN and exercise tolerance. In other words, the lower the exercise tolerance of the body, the more significantly the BUN level increases [40]. Therefore, the accumulation of serum LA, LDH, and BUN could illustrate the speed and degree of fatigue development as well as the workload intensity. As expected, exhaustive swimming significantly elevated the levels of LA, LDH, and BUN in serum, while these increases were dramatically attenuated in the LN-treated groups (Table 2). This indicated that LN could enhance the body's exercise endurance by reducing the metabolism of energy and protein.

Exercise has various effects on skeletal muscle and hepatic function, enhancing both nutrient metabolism and antioxidant capacity. Accumulating evidence indicates that exhaustive exercise could injure liver cells by decreasing blood flow in the liver [41] and the portal vein [42], which often causes hypoxia of hepatocytes, eventually inducing their necrosis. Strenuous physical exercise leads to increased production of reactive oxygen species, which facilitates blood vessel dilation so as to increase blood flow and the supply of oxygen and glucose to working skeletal muscles [43]. However, exhaustive exercise may impair the cellular function of the skeletal muscle by affecting its integrity and thus is considered to be responsible for muscle fatigue during exercise. Compared with those in the MC group, the swelling of the liver and skeletal muscle cells and the degree of inflammatory cell infiltration were significantly reduced in the LN-treated groups (Figure 3). Meanwhile, acute physical exercise can result in the shrinkage of liver cells, and the volume change of the liver associated with this is linked not only to a decrease in hepatic glycogen level but also to liver dysfunction [44]. Therefore, elevations of ALT and AST are predictors of liver cell damage and are characteristic responses to exhaustive physical exercise [45, 46]. Here, we found significant increases in the levels of ALT and AST in the serum in response to chronic fatigue-induced liver dysfunction, which were attenuated in the LN treatment groups (Table 2).

Oxidative stress involves an imbalance between the production of ROS and the ability of the antioxidant defense system (i.e., antioxidant enzymes) to scavenge them [47]; it has been implicated in both chronic fatigue and fatigue-related disorders. Under normal conditions, regular physical exercise helps the body to function better, but exhaustive exercise may result in excessive ROS in vivo, leading to lipid peroxidation of the membrane structure and oxidative damage to cells in target tissues such as liver and skeletal muscles [48, 49]. MDA is an intermediate product of lipid peroxidation, which is one of the major outcomes of free radical-mediated injury. Exhaustive exercise can increase the ROS and MDA levels in the liver and muscle of rats and mice [50, 51]. Enzymatic and nonenzymatic antioxidants are two major defense systems that work to reduce the harmful effects of ROS and MDA on cells [52]. Antioxidant enzymes such as SOD and GSH-Px are regarded as the principal components of the enzymatic antioxidant defense systems to help fight against fatigue and protect cells by reducing the generation of active oxygen radicals and breakdown products of metabolization from oxidative damage [53]. Exhaustive exercise increases in ROS and MDA were significantly attenuated by LN treatment. Similar kinetics were observed for SOD and GSH-Px in response to weight-forced exercise, which were almost completely reversed by LN treatment in liver and muscle tissues (Figure 4). Therefore, our results indicate that LN has an effective antioxidant activity that prevents lipid peroxidation and protects cells from oxidative stress injury in the liver and skeletal muscle of rats after exhaustive exercise.

Inflammation is a condition that arises in response to exercise-mediated oxidative stress among others. TNF-*α*, IL-1*β*, and IL-6 are three proinflammatory cytokines; the excessive release of which significantly limits physical performance under conditions of intense exercise [54–56]. This indicates the close association between cytokine responses and the pathogenesis of oxidant stress and organ dysfunction induced by chronic fatigue [57]. Prior studies showed that TNF-*α*, along with IL-1*β* and IL-6, induces the synthesis of neurotransmitters, which is associated with increased fatigue and ill health in previously healthy subjects [58, 59]. IL-10 is an anti-inflammatory cytokine that can negatively modulate the activity of TNF-*α*, IL-1*β*, and IL-6 and is used as a marker of the defensive capacity of the body in inflammatory responses [60]. Here, we found significant increases in the levels of proinflammatory cytokines (TNF-*α*, IL-1*β*, and IL-6) in the liver and skeletal muscle in rats after exhaustive exercise, which were attenuated in the LN treatment groups (Figures 5(a), 5(b), and 5(c)). Similarly, IL-10, the anti-inflammatory cytokine, was downregulated in the MC group; this effect was also ameliorated by LN treatment (Figure 5(d)). This suggested that the protective effects of LN on the liver and skeletal muscle reflected in the pathologic changes detailed above may result from its anti-inflammatory activity.

To clarify the molecular mechanism by which LN is involved in the fatigue and fatigue-related organ dysfunction induced by exhaustive exercise, we evaluated the expression of oxidative stress-related signaling genes in liver and skeletal muscle tissues. Increasing evidence has established that exhaustive exercise can induce fatigue and fatigue-related oxidative damage in organs. Heme oxygenase-1 (HO-1), an inducible and rate-limiting enzyme, appears to catalyze the degradation of heme to carbon monoxide and biliverdin, which is subsequently converted to bilirubin (a powerful antioxidant) by biliverdin reductase [61]. Studies have shown that HO-1 activation contributes to inhibiting the generation of proinflammatory cytokines such as TNF-*α*, IL-1*β*, and IL-6 in pathophysiological conditions [62–65] and it has emerged as a key molecule for regulating inflammatory responses and oxidative stress in a variety of diseases, such as chronic fatigue syndrome, diabetes mellitus, hepatitis, and cancer [66]. In addition, HO-1 is one of the phase II detoxification enzymes induced by the antioxidant response element- (ARE-) mediated gene, and its expression is modulated by a transcription factor, nuclear factor erythroid 2-related factor 2 (Nrf2). Nrf2 is normally present in the cytoplasm and binds to kelch-like ECH-associated protein 1 (Keap1) as a redox-sensitive master regulatory transcriptional factor. However, when oxidative stress occurs in cells, Nrf2 dissociates from Keap1 and then binds to ARE in the promoters of phase II antioxidant enzyme genes (GST, HO-1), thereby controlling their expression [67]. In other words, Nrf2 induces the expression of antioxidant enzymes such as HO-1 by binding to ARE in the promoters of these genes [68]. Therefore, the Nrf2/ARE pathway is thought to be an important target for the therapy of oxidative stress-induced diseases. In our study, RT-PCR and Western blot analysis were performed to determine whether LN enhances HO-1 and Nrf2 expression. We found that exhaustive swimming led to significant reductions in HO-1 and Nrf2 levels, which were normalized by LN treatment (Figures 6 and 7). Therefore, LN treatment can enhance nuclear Nrf2 and HO-1 protein expression in the liver and skeletal muscle. These findings suggested that the Nrf2/ARE pathway is an underlying molecular mechanism of the therapeutic effects of LN and further research is needed to explore the possibilities of which as a therapeutic intervention for the prevention of major organ dysfunction induced by exhaustive exercise.

## 5. Conclusions

Here, all the results demonstrated that the administration of LN could significantly improve the endurance of rats in the FST. Moreover, LN clearly prevented the dysfunction of the liver and skeletal muscle induced by oxidant stress by regulating inflammatory and oxidative reactions. The underlying mechanisms responsible for the antifatigue effect of LN involves mainly the modulation of oxidative stress and inflammation by activating the Nrf2/ARE pathway. These results suggest that LN has significant health benefits due to its antifatigue activity, which provided scientific evidence for further development of natural products for prevention and treatment of diseases related to fatigue.

## Figures and Tables

**Table 1:** Sequence of primers used for the RT-PCR assays.

Genes	Primer sequences	
	Forward primer (5′ → 3′)	Reverse primer (5′ → 3′)
Nrf2	TTCCTCTGCTGCCATTAGTCAGTC	GCTCTTCCATTTCCGAGTCACTG
HO-1	CTGGAAGAGGAGATAGAGCGAA	TCTTAGCCTCTTCTGTCACCCT
*β*-actin	GCCATGTACGTAGCCATCCA	GAACCGCTCATTGCCGATAG

**Table 2:** Effects of LN on serum biochemical parameters and glycogen storage after exhaustive swimming.

Group	Dose (mg/kg)	BUN (IU/L)	LDH (IU/L)	LA (mmol/L)	LG (mg/g)	MG (mg/g)	AST (U/L)	ALT (U/L)
NC	—	8.9 ± 0.3	128.5 ± 3.7	8.9 ± 0.4	23.4 ± 1.5	1.35 ± 0.17	121.23 ± 18.65	36.76 ± 4.76
MC	—	16.3 ± 1.5^##^	256.8 ± 16.3^##^	30.4 ± 1.0^###^	7.9 ± 1.3^###^	0.74 ± 0.09^##^	335.66 ± 62.88^###^	99.44 ± 31.34^###^
AA100	100	11.7 ± 0.6^∗∗^	138.1 ± 12.1^∗∗∗^	12.5 ± 1.2^∗∗∗^	14.7 ± 0.4^∗^	1.16 ± 0.09^∗∗^	168.94 ± 21.37^∗∗^	47.32 ± 6.68^∗^
LN75	75	9.4 ± 0.7^∗∗^	139.2 ± 6.9^∗∗∗^	10.9 ± 1.6^∗∗∗^	16.0 ± 0.7^∗∗^	1.12 ± 0.15^∗∗^	137.56 ± 15.42^∗∗^	40.55 ± 11.07^∗∗^
LN50	50	10.2 ± 0.9^∗∗^	177.9 ± 18.3^∗^	11.3 ± 0.8^∗∗∗^	13.2 ± 0.4^∗^	1.05 ± 0.13^∗^	196.17 ± 35.29^∗∗^	50.27 ± 7.81^∗^
LN25	25	11.1 ± 0.7^∗∗^	201.5 ± 22.6^∗^	16.4 ± 1.1^∗∗^	10.8 ± 0.2^∗^	0.98 ± 0.07^∗^	224.95 ± 41.16^∗^	67.32 ± 14.65^∗^

Data are means ± SD (*n* = 10). *^##^P* < 0.01 and *^###^P* < 0.001 compared with NC (normal control group); ^∗^*P* < 0.05, ^∗∗^*P* < 0.01, and ^∗∗∗^*P* < 0.001 compared with MC (model control group). BUN: blood urea nitrogen; LDH: lactate dehydrogenase; LA: serum lactic acid; LG: liver glycogen; MG: skeletal muscle glycogen; AST: aspartate aminotransferase; ALT: alanine aminotransferase; AA100: positive control group (exhaustive swimming rats treated with ascorbic acid); LN75: LN high-dosage group (exhaustive swimming rats treated with LN at 75 mg/kg); LN50: LN medium-dosage group (exhaustive swimming rats treated with LN at 50 mg/kg); LN25: LN low-dosage group (exhaustive swimming rats treated with LN at 25 mg/kg).
